# Lessons learned of emerging Chikungunya virus in two populations of social vulnerability of the Colombian tropics: epidemiological analysis

**DOI:** 10.1186/s13690-018-0284-2

**Published:** 2018-07-23

**Authors:** Misael Oviedo-Pastrana, Nelson Méndez, Salim Mattar, Germán Arrieta, Luty Gomezcaceres

**Affiliations:** 1 Universidad de Córdoba, Instituto de Investigaciones Biológicas del Trópico, Carrera 6 # 76-103, Montería, Cordoba, Colombia; 2 Corporación Universitaria del Caribe (CECAR), Grupo de Salud Pública, Km 1, vía Corozal, Sincelejo, Colombia; 3 Clínica Salud Social SAS, Carrera 16 # 27A-74, Sincelejo, Colombia; 4 Facultad de Medicina Veterinaria y Zootecnia, Universidad de Córdoba Universidad de Córdoba, Instituto de Investigaciones Biológicas del Trópico (IIBT), Carrera 6 # 76-103, Montería, Cordoba, Colombia

**Keywords:** Epidemiology, Public health systems, Cullicidae

## Abstract

**Background:**

Notwithstanding the strong epidemiological impact of the Chikungunya in the Colombian Caribbean, in 2014, not the entire population were affected in the same way. This study describe the demographic, socio-economic, clinical and epidemiological aspects of the de Chikungunya in Ovejas and Corozal, two neighboring municipalities with high vulnerability in health in the Colombian Caribbean.

**Methods:**

A cross-sectional study was performed in February 2015. A convenience sampling was carried out in 971 families affected with chikungunya. Also, a socio-demographics, clinical and epidemiological questionnaire was carried out for people who met the definition of suspected Chikungunya clinical case. For the statistical analysis, data and variables, frequencies, proportions and means were compared in the two municipalities studied. A logistic regression model was constructed to explain the effect of factors studied on the risk of family infection (RFI) or likelihood of contagion within each household. Was used the software EpiInfo 7.2.2.2 and a significance level with *p*-value < 0.05.

**Results:**

In Ovejas, 516 households were affected by Chikungunya, 48% (1269/2631) of their inhabitants became sick; in Corozal, 455 families were affected and 42% (839/1999) of their members became sick. The evolution of the epidemic curves of Chikungunya outbreak was different in the two studied areas, the disease was more aggressive in Ovejas. Ten variables were pre-selected by univariate analysis to explain the RFI by Chikungunya, and were integrated into a logistic regression model. The final model was constructed with the following variables: municipality, gender, occupation, family income, use of repellent and fumigation. The logistic model was assessed as appropriate; however, the biases in the selection of the surveyed dwellings and in the selection of symptomatic patients could influence the results.

**Conclusions:**

It was demonstrated the epidemiological complexity of Chikungunya and the serious problem caused in populations with high vulnerability in health. The accurate association observed in the logistic regression model suggests the role of the factors studied as determinant in the rate of infection of the Chikungunya; coverage problems and surveillance in health care, demographic aspect, socio-economic problems and lack of preventive measures could explains the risk of family infection by Chikungunya in some areas tropics of Colombia.

**Trial registration:**

number approval 007–2016 ethics committee-IIBT.

## Background

The Chikungunya is an emerging arbovirus in the Americas; it is an Alphavirus belonging to the family Togaviridae. Chikungunya virus (CHIKV) causes disease via direct spillover as well as by entering a human–mosquito–human cycle in urban areas, typically involving transmission by the anthropophilic mosquito *Aedes aegypti* and recently also by *Ae. albopictus*; this leads to major epidemics involving millions of persons with efficient spread via infected air travelers during recent outbreaks [1]. CHIKV is the second most widely distributed arboviral disease after dengue fever. In total, 106 countries/territories have reported autochthonous vector-borne transmission of the disease [2]. The disease is widespread in most of the Americas: of the 52 countries/territories found suitable for the vectors, 46 have reported autochthonous vector-borne transmission of the disease [2]. In December 2013 on the Caribbean island of Saint Martin, the first autochthonous transmission of Chikungunya virus was reported [3, 4]. Currently, two CHIKV genotypes have been reported in Americas; the Asian genotype involved in the Colombian outbreak and the East/Central/South African Genotype (ECSA) detected in Brazil [5, 6].

In the Americas, in 2015, the Pan American Health Organization (PAHO) reported 730,969 cases of CHIKV, of which 49.15% corresponded to Colombia, with an incidence of 725.4 cases per 100,000 inhabitants [7]. In 2016, there were 496,850 cases of CHIKV, the countries that most reported were Brazil (83%), Bolivia (4.4%) and Colombia (3.9%); the incidences per 100,000 inhabitants were: Brazil with 196.82, Bolivia with 201.83 and Colombia with 40.21 [8]. In 2017, 184,700 cases of CHIKV were reported in the Americas, of which 1080 cases were reported in Colombia, with an incidence of 2.20 per 100,000 inhabitants [9].

In Colombia, CHIKV infection created a new epidemiological stage, the first autochthonous transmission of CHIKV occurred in September 2014 in the population of Mahates, Bolivar [10]; three months later, in epidemiological week 53, 96,687 cases were informed in the country, of which 66,118 (68.4%) belonged to the Caribbean region and 13,464 (13.9%) corresponded to the Sucre department [11].

Although the disease caused by CHIKV is rarely fatal, it represents a worrisome public health problem due to the economic and social impact associated with extensive morbidity in all age groups. In addition, the presentation of chronic arthralgia may persist for years and the development of severe neurological complications in newborns infected during birth and in the elderly with underlying medical conditions [12]. All this implies expenses in the health care and in medicines to treat the symptoms of the disease, in addition, the infection by the CHIKV produces labor incapacities and therefore economic losses due to the absence of work [12].

In Colombia, the intervention strategies against CHIKV have been adjusted to World Health Organization (WHO) prevention and control guidelines [13], focusing on the reduction of mosquito breeding sites, insecticide application and community education with an emphasis on the prevention of each disease familial.

Regardless the strong epidemiological impact of the Chikungunya in the Colombian Caribbean not all populations were affected in the same way [14, 15]. Socio-economic and demographic aspects could be the main cause of the disproportionate effect of the disease even among populations that are very close and with high vulnerability in health; this study tries to demonstrate these effects. The objective of this study was to describe the demographic, socio-economic, clinical and epidemiological aspects of the Chikungunya outbreak in two neighboring populations with high health vulnerability in the Colombian Caribbean.

## Methods

### Study area

**Fig. 1:**
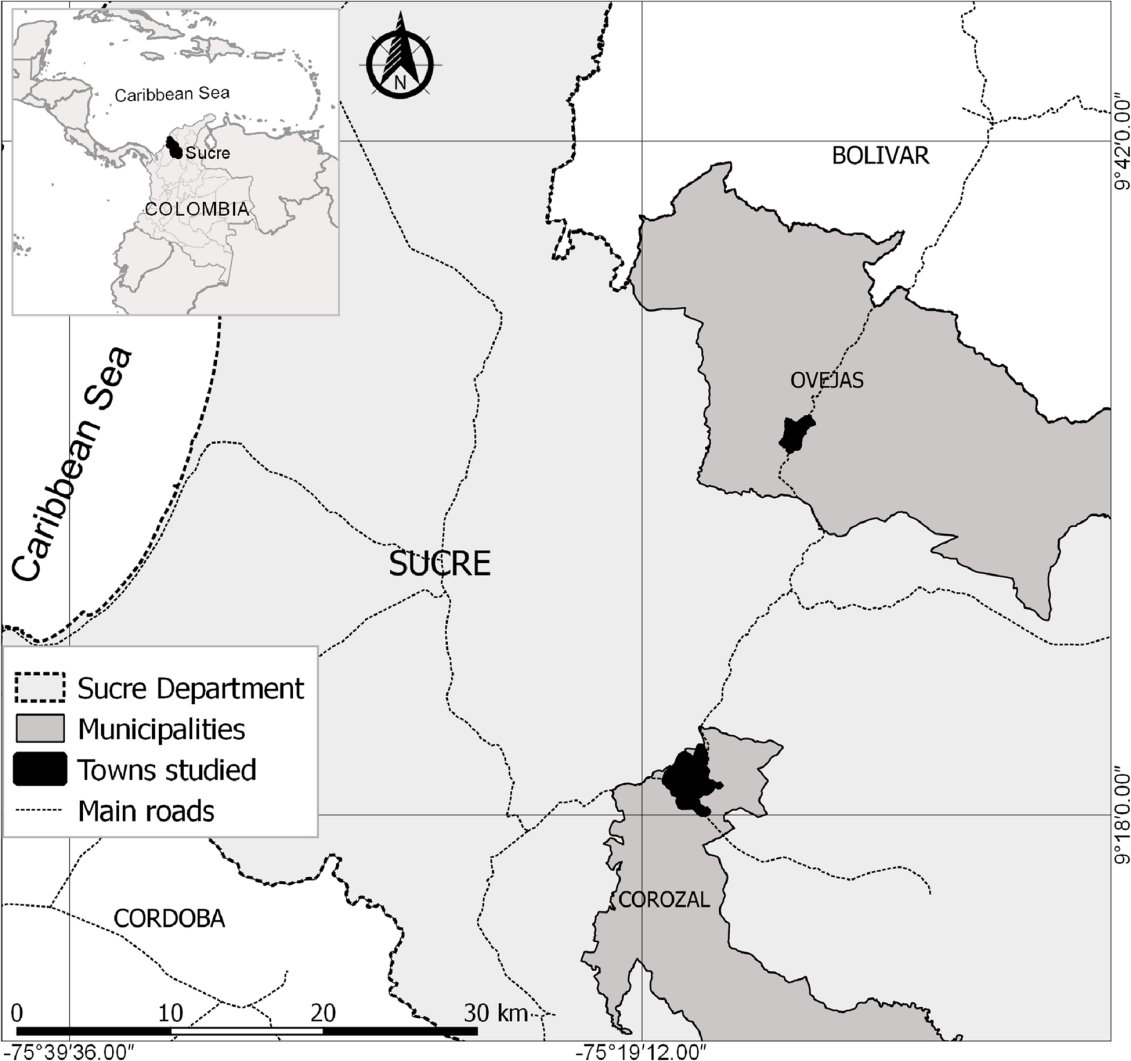
Study area in the towns of Corozal and Ovejas, in the department of Sucre, Colombia

The study was carried out in the urban area of the municipalities of Corozal and Ovejas, in the department of Sucre, Colombia (Fig. 1). The populations of Ovejas and Corozal are separated by 22 km and are communicated by a route of high traffic and commerce; both municipalities are in the tropics and during the year presents a dry season (December–April) followed by rainy season (May–November), however, the El Niño phenomenon could be affected this seasonality. The population of Ovejas and Corozal has an average temperature of 28 °C and an altitude of 265 and 174 m above sea level, respectively. In 2015, the urban population of Ovejas and Corozal municipalities was estimated at 11,947 and 51,157 inhabitants, respectively [16]. These areas were selected due to its proximity and because both populations showed an emerging CHIKV outbreak at the end of 2014. In addition, both are relatively near to the population of Mahates, where probably the first autochthonous case of this disease appeared in the country [10].

### Type of study, population and sampling

A cross-sectional study was conducted in February 2015 to describe the demographic, socio-economic, clinical and epidemiological characteristics of the population in two neighboring municipalities affected by a Chikungunya outbreak. A convenience sampling was carried out in the two chosen populations, selecting areas of low socio-economic level and favorable environmental conditions for mosquitoes proliferation, which include proximity to water reservoirs and the accumulation of garbage; the selected sites were delimited by previous knowledge, guidance of the Municipal Health Secretary and simple observation. At each of the selected sites, all households were visited and selected only if anyone of the family members had Chikungunya in the previous months (September/2014 to February/2015). Empty homes were not evaluated. Responsible adults (often parents and grandmothers) present in each dwelling provided the data requested in the survey. In each selected dwelling detailed information was taken of all sick people, no information was obtained from people who were not sick. Detailed information of children and persons absent during the interview was provided by responsible adults present. Eight people joined applying the survey, all of them were under previous training.

The clinical description revealed by the people in the interview in each home matched with the clinical definition of a suspected CHIKV clinical cases of the Colombian National Institute of Health [17], by the way, many of these people received medical attention and a clinical diagnostic. A clinically suspected case was defined as a person who had a fever greater than 38 °C, severe arthralgia or acute onset arthritis and multiform erythema, typical rash or symptoms not explained by other medical conditions, also the person who lived or visited a municipality where there was evidence of Chikungunya virus circulation or a person located in a municipality with a radius of 30 km to municipalities with viral circulation.

A socio-demographics, clinical and epidemiological questionnaire was implemented for people who met the definition of suspected CHIKV clinical case in each selected household. Relevant aspects of the questionnaire included: Characteristics of the inhabitants (total of inhabitants, sick people, sex, age, occupation and family income), medical consultation, clinical aspects, medicines used, public services (aqueduct, storage of water in tanks, garbage collection) and preventive aptitudes for reduce the proliferation and bites of mosquitoes (Fumigation and uses of repellents and mosquito nets). Possible cases of onset of symptoms of the disease and other clinical aspects (clinical, symptomatic and therapeutic description) were questioned.

### Statistical analysis

For the statistical analysis, data and variables, frequencies, proportions and means were compared in the two municipalities studied. We also determined and compared the temporal distribution of cases of Chikungunya.

A logistic regression model was constructed in the dataset to explain the effect of demographic, socio-economic, and behavioral factors on Chikungunya infection in affected households. The variable response expressed the risk of family infection (RFI) or likelihood of contagion within each household. To obtain the RFI the quotient was determined between the number of individuals affected with Chikungunya and the total number of inhabitants within each household. Two categories of RFI (high risk and low risk) were determined using the respective median as cutoff point. The explanatory variables that participated in the construction of the model were pre-selected according to the univariate statistic using the chi-square test. For the pre-selection of variables and for the construction of the model we used a significance level with *p*-value < 0.05. Hosmer-Lemeshow-C, Hosmer-Lemeshow H, and Cessie & Van Houwelingen test were used to evaluate the logistic model [18, 19]. Analyzes were performed using software EpiInfo, version 7.2.2.2.

## Results

During the outbreak, 971 households inhabited by 4630 people, were affected by Chikungunya. A total of 2108 people were studied, 45.82% met the clinical case definition for Chikungunya. Table 1 shows a comparative summary of the variables studied in each municipality, 516 households were affected in the population of Ovejas, 48% (1269/2631) of their inhabitants became sick; 455 households were affected in the population of Corozal, 42% (839/1999) of their members became sick. Women were the most affected by CHIKV in both municipalities, 29% (765/2631) in Ovejas and 26.6% (533/1999) in Corozal. The other frequencies described in Table 1 are related to the inhabitants affected with Chikungunya in each municipality.

**Table 1:** Comparative summary of the variables studied in the municipalities of Ovejas and Corozal, Sucre - Colombia

Variables		Ovejas				Corozal			
	Category	*N*	%	Range	Mean	*N*	%	Range	Mean
Households	Homes	516	100			455	100		
Total Inhabitants	People in houses	2631	100	1–16	6	1999	100	1–16	5
Affected inhabitants	People with CHIKV	1269	48	1–9	4	839	42	1–8	3
Gender	Female	765	29			533	26.6		
	Male	504	19			306	15.4		
Duration of symptoms	Sick days	–	–	1–150	21	–	–	1–150	37
Medical consultation	Medical care	655	51.6			384	45.8		
	Hospitalization	57	4.5			62	7.4		
	Days hospitalized	–	–	1–3	1.8	–	–	1–30	2.5
	Sub-registration	614	48.4			455	54.2		
Aftermath	Polyarthralgias	243	19.2			350	41.7		
Expenses	Expenses for CHIKV	1119	88.2	1–666	24.1	513	61.1	1–533	29.3
Family income	> minimum salary	169	13			110	13		
	≤ minimum salary	1100	87			729	87		
	Total income	–	–	10–833	152	–	–	21–666	118.7
Age	Young (< 15 years old) old old)	174	14			110	13		
	Adult (15–60 years old)	908	72			564	67		
	Elder (> 60 years old)	187	15			165	20		
Occupation	Housewife	15	1.2			329	39.2		
	Student	258	20.3			189	22.5		
	Employee	259	20.4			207	24.7		
	Unemployed	681	53.7			99	11.8		
	Pensioned	56	4.4			15	1.8		
Scholarship	None	254	20			67	8		
	Primary	387	30			276	33		
	High School	529	42			343	41		
	Technical University	99	8			153	18		
Preventive measures	Cleaning service	672	53.0			838	99.9		
	Water tanks	1257	99.1			464	55.3		
	Stagnant water	79	6.2			168	20.0		
	Repellents	166	13.1			190	22.7		
	Fumigations	272	21.4			372	44.3		
	awning	44	3.5			13	1.6		
	insect screen	12	1.0			10	1.2		

In the population of Corozal, the average duration of the Chikungunya was 37 days, 45.8% of those affected received medical care and 7.4% were hospitalized. In the population of Ovejas, the average duration of the disease was 21 days, 51% of those affected received medical care and 4.5% were hospitalized. Polyarthralgia was the predominant result and its percentage in the population of Corozal doubled to population of Ovejas. 88% of the people affected in the population of Ovejas had to afford the expenses of the disease and 61% in the population of Corozal; mean of 24.1 dollars in Ovejas and 29.3 dollars in Corozal. The monthly income in the affected families of the two municipalities was quite low, 152 dollars in Ovejas and 118 dollars in Corozal. In the population of Ovejas there was a reduction in the use of preventive measures.

**Fig. 2:**
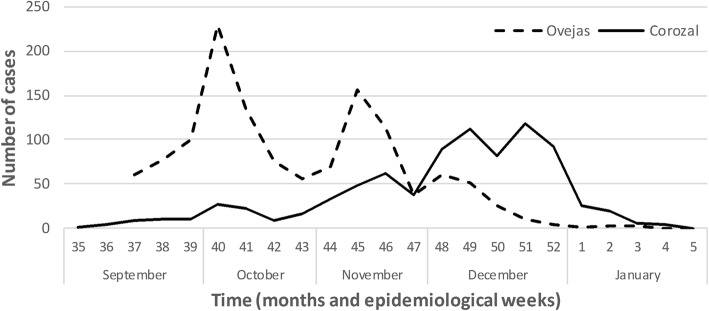
Temporal distribution of Chikungunya cases in the municipalities of Ovejas and Corozal, Sucre-Colombia

The difference between the start dates of the disease in the two populations was 10 days, in Corozal started on 05/09/2014 and in Ovejas on 15/09/2014. The temporal distribution of the Chikungunya outbreak in the two populated areas is shown in Fig. 2. The two epidemic curves presented different distribution. In the municipality of Ovejas the disease was more aggressively established with 61 cases in week 37, and the spread had a high variation, with epidemic peaks at week 40 and 45. In contrast, in Corozal the disease was milder with 9 cases in week 37 and its evolution had little variation during the first months, however, between November and December the number of cases increased considerably, with epidemic peaks in the Week 49 and 51. The decline of the epidemic in the two populations was in January 2015.

The most frequent symptoms of Chikungunya in the 2108 inhabitants affected in the two localities studied were fever, arthralgia, rash and headache (Table 2); however, using the Odds Ratio criterion, the clinical effect of the disease showed significant differences. The results showed that the inhabitants of the population of Ovejas had greater presentation of the symptoms compared to the inhabitants of Corozal. The greater presentation of symptoms in the population of Ovejas entailed a higher attendance in the health centers, being Odds Ratio 1.26 times greater in relation to the inhabitants of the population of Corozal.

**Table 2:** Comparison of the main clinical aspects caused by Chikungunya in the municipalities of Ovejas and Corozal, Sucre - Colombia

Clinical aspects	Ovejas		Corozal		OR	95% CI		*p*-value
	*n*	%	*n*	%		inf	sup	
Fever	1194	94.1	728	86.8	2.43	1.78	3.30	< 0.0001
Arthralgia	1167	92.0	681	81.2	2.65	2.03	3.46	< 0.0001
Rash	972	76.6	568	67.7	1.56	1.28	1.89	< 0.0001
Cephalea	783	61.7	434	51.7	1.50	1.26	1.79	< 0.0001
Medical consultation consultadvisor	655	51.6	384	45.8	1.26	1.06	1.50	< 0.0001

The people who received medical attention were the oldest, with an average of 40 years; there was a predominance of women (65%) and the duration of the symptoms had an average of 31 days. The people who did not receive medical attention had an average of 35.6 years, 58% were women and the duration of the symptoms had an average of 23 days. These differences were similar in the two municipalities studied. Non-medical attention shows the under-registration of the disease in the two populations, 48.4% in Ovejas and 54.2% in Corozal.

Regarding medication use, the most used in both municipalities were acetaminophen, nimesulide, naproxen, diclofenac, loratadine, ibuprofen, piroxicam and dipyrone (Table 3). The low medical consultation (Table 2) and the high use of acetaminophen (Table 3) observed in both municipalities suggest a high rate of self-medication in people who did not attend the health center. However, although the greater clinical presentation of the disease was in the population of Ovejas, this population used fewer drugs compared to the population of Corozal. The OR was always significant and indicative of protection, expressing in this case a lower use of drugs in the population of Ovejas. The use of repellents was also lower in the population of Ovejas, approximately, 2 times smaller (1/0.51 OR) in relation to the population of Corozal.

**Table 3:** Comparison of the main drugs used against Chikungunya in the urban areas of Ovejas and Corozal, Sucre - Colombia

Drugs	Ovejas		Corozal		OR	95% CI		*p*-value
	*n*	%	*n*	%		inf	sup	
Acetaminophen	942	74.2	747	89.0	0.35	0.27	0.45	< 0.0001
Nimesulide	157	12.4	189	22.5	0.48	0.38	0.61	< 0.0001
Naproxen	94	7.4	240	28.6	0.20	0.15	0.26	< 0.0001
Diclofenac	84	6.6	104	12.4	0.50	0.37	0.68	< 0.0001
Loratadine	64	5.0	148	17.6	0.25	0.18	0.34	< 0.0001
Ibuprofen	39	3.1	118	14.1	0.19	0.13	0.28	< 0.0001
Piroxicam	32	2.5	65	7.7	0.31	0.20	0.47	< 0.0001
Dipyrone	19	1.5	47	5.6	0.25	0.14	0.44	< 0.0001
Repelents	166	13.1	190	22.6	0.51	0.40	0.64	< 0.0001

Table 4 shows a statistical summary of the RFI by Chikungunya for the total data set and for the two populations studied. The probability of infection was higher than 0.60 was considered as high RFI and the lowest as low RFI. The RFI by Chikungunya in the municipality of Ovejas had a mean of 0.64 and a median of 0.70; 32.9% of households including 51.7% of individuals had high RFI. The RFI by Chikungunya in the population of Corozal had a mean of 0.59 and a median of 0.60; 28.1% of households with 41.6% of individuals had high RFI.

**Table 4:** Risk of family infection by Chikungunya in the municipalities of Ovejas and Corozal, Sucre - Colombia

Municipality	RIF												
	Statistical summary						households			People			
		%						(%)				(%)	
	mean	min.	25	50	75	max.	*n*	High	Low	am	n	High	Low
Ovejas	0.64	0.1	0.4	0.7	1.0	1.0	516	32.9	67.1	1269		51.7	48.3
Corozal	0.59	0.1	0.3	0.6	0.8	1.0	455	28.1	71.9	839		41.6	58.4
Total	0.62	0.1	0.4	0.6	0.9	1.0	971	30.7	69.3	2108		47.7	52.3

Of the variables studied to explain RFI by Chikungunya in the total dataset, ten were pre-selected by univariate analysis (Table 5). Municipality, gender, age, occupation, household income, water stored in tanks, toilet (garbage collection), mosquito net, repellent and fumigation were the pre-selected variables.

**Table 5:** Pre-selected variables related to RFI by Chikungunya in the most vulnerable households in the urban areas of Ovejas and Corozal, Sucre - Colombia

Demographic, socioeconomic and preventive factors	Category	Low rate		High rate		OR	95% CI		*p*-value
		n	%	n	%		Lower	Upper	
Municipality	Corozal	490	58.4	349	41.6	1.50	1.26	1.79	0.000
	Ovejas	613	48.3	656	51.7				
Gender	Female	728	56.1	570	43.9	1.48	1.24	1.76	0.000
	Male	375	46.3	435	53.7				
Aged	Elderly (> 60 years)	188	53.4	164	46.6				
	Adult (15–60 years)	803	54.5	669	45.5				0.000
	Young (< 15 years)	112	39.4	172	60.6				
Occupation	Other	931	56.0	730	44.0	2.04	1.65	2.52	0.000
	Student	172	38.5	275	61.5				
Familiar income	> minimum salary	177	63.4	102	36.6	1.69	1.30	2.19	0.000
	≤ minimum salary	926	50.6	903	49.4				
Stored water	No	226	58.4	161	41.6	1.35	1.08	1.69	0.008
	Yes	877	51.0	844	49.0				
Garbage recollection	No	261	43.7	337	56.3	0.61	0.50	0.74	0.000
	Yes	842	55.7	668	44.3				
Mosquito nets	No	1086	52.1	1000	47.9	0.31	0.11	0.86	0.019
	Yes	17	77.3	5	22.7				
Repellent	No	867	49.5	885	50.5	0.49	0.39	0.63	0.000
	Yes	236	66.3	120	33.7				
Fumigation	No	707	48.3	757	51.7	0.58	0.48	0.70	0.000
	Yes	396	61.5	248	38.5				

Among the demographic factors, being a resident of the population of Ovejas was 1.50 times more predisposing to high risk of family infection in relation to the inhabitants of Corozal. In general, the families of the populations of Ovejas and Corozal had an increased risk of infection. However, high risk of family infection was observed in families with greater presence of males (OR: 1.48) and in families with a greater presence of students (OR: 2.04); people < 15 years also contributed significantly.

From a socio-economic point of view, the lower income families than the minimum wage (257.48 USD, USD 1 = 2392.46 Colombian pesos to December 31, 2014) presented higher RFI (OR = 1.69) in relation to the families with higher incomes. Also, irregular water storage for domestic use was associated with high RFI (OR = 1.35), which contributes to the proliferation of mosquitoes inside houses. On the other hand, the collection of garbage by the responsible entity was related to a low RFI (OR = 0.61). Mosquito nets, repellent and fumigation were variables directly related to the prevention, reduction and/or elimination of vectors; their uses would constitute a low RFI; their respective ORs were: 0.31, 0.49 and 0.58.

The pre-selected variables were integrated into a logistic regression model (Table 6). Different interactions by stepwise selection methods were evaluated until obtaining a statistically consolidated model to explain the high rates of family infection by Chikungunya in the total data set. The final model was made up of the following variables: municipality, gender, occupation, family income, use of repellent and fumigation. Hosmer-Lemeshow-C (chi-square = 10.363, *p*-value = 0.1689), Hosmer-Lemeshow H (chi = 12.805, p-value = 0.1187) and Cessie & Van Houwelingen test (z = − 0.6946; *P*-value = 0.4873) assessed fit the model as appropriate. However, the biases in the selection of the surveyed dwellings and in the selection of symptomatic patients could influence the results.

**Table 6:** Final logistic model to explain RFI by Chikungunya in the most vulnerable households in the municipalities of Ovejas and Corozal, Sucre – Colombia

Variables	Categories	Odds Ratio	95% CI		*P*-Value
			Lower	Upper	
Municipalities	Ovejas / Corozal	1.358	1.127	1.636	< 0.0013
Gender	Male / Female	1.398	1.166	1.675	< 0.0003
Occupation	student / Other	1.943	1.561	2.419	< 0.0001
Family income	≤ / > minimum salary	1.394	1.062	1.831	< 0.0167
Repellents	Yes / No	0.642	0.499	0.826	< 0.0006
Fumigations	Yes / No	0.691	0.564	0.845	< 0.0003

## Discussion

The Chikungunya virus spread during the second half of 2014 in the Colombian Caribbean, producing a serious public health problem. This study analyzes an outbreak of CHIKV in populations with high health vulnerability, in the municipalities of Ovejas and Corozal, in the Colombian Caribbean. The study shows that despite the spatial proximity, similar environmental conditions, presence of the same vector and the same etiologic agent on inhabitants without previous exposure, the clinical and epidemiological behavior of the disease had a different evolution. It is possible that the preventive attitudes and socio-economic and different demographic characteristics of the towns influence the epidemiology of transmission patterns.

The first autochthonous case of Chikungunya in the country reported by the National Institute of Health was in San Joaquín, municipality of Mahates in the department of Bolivar [10] located 90 km from the population of Ovejas and 112 km from the municipality of Corozal. In the present study, the outbreak of Chikungunya in the municipalities of Ovejas and Corozal began in early September, however, the virus may have previously circulated silently in other localities close to the municipalities studied. In fact, there is a report of a first clinical case of Chikungunya in the municipality of Ovejas on August 1, 2014 [20], 42 days before the official notification of the National Institute of Health (INS for its acronym in Spanish), which means that the outbreak probably began before official notification. In the municipality of Corozal, some suspicious CHIKV cases were also identified prior to official health notification.

The apparent presence of suspected Chikungunya cases, prior to the date of health official notification, reflects weaknesses in the local public health system, mainly in the prevention, notification, diagnosis and control of vectors; in addition, a low perception of risk, ignorance of the disease [10] and consequently under-registration of cases [21, 22]. A silent and previous circulation of Chikungunya could reduce the infection rate in some families studied.

A more aggressive epidemiological evolution of the Chikungunya epidemic curves in the municipality of Ovejas presented similar results with the number of cases reported by the National Institute of Health in the year 2014, the attack rate in Ovejas was found twice as high as that of Corozal [23]. The strong presence of the Chikungunya virus in the town of Ovejas and its high attack rate could be a direct consequence of the epidemiological risk factors studied and the failures in the municipal epidemiological surveillance system, because the disease could have circulated in silence for 42 days before official notification.

On the other hand, it is notable the fact of finding differences in the risk of infection within areas with high vulnerability in health. The differences found between low rate of RFI and High rate of RFI could be direct consequence of a greater localization of vulnerable families in the municipality of Ovejas; a greater presence of men, students and children < 15 years; a lower family economic capacity and a greater deficiency in the establishment of measures for the prevention and control of mosquitoes.

However, in the two populations studied, it was observed that many patients did not go to health care centers, the sub-notification in the municipality of Ovejas was 48.4% and in Corozal, 54.2%. sub-notification may be due to a favorable course of the disease, and self-medication or lack of preparation to assist the physician due to overcrowding and collapse in the provision of health care services [22]. As in the two municipalities, 87% of those affected had a family income below the minimum income ($ 257.48 USD).

In the present study fever, arthralgia, rash and headache were the major clinical manifestations of Chikungunya although these were also observed in other studies [10, 24]. However, the clinical manifestations were greater in the inhabitants of the municipality of Ovejas than in Corozal. These data are in concordance with the highest attack rate in the town of Ovejas, the most aggressive epidemiological evolution and the highest number of health center assistance. In contrast, with less drug use in the municipality of Ovejas, the inhabitants of Corozal used medications in greater quantity. Increased consumption of medicines in the population of Corozal could be due to a greater offer by the health entities in relation to that of the population of Ovejas. In addition, as the population of Corozal doubled the presentation of sequelae, this could also encourage the greater consumption of medicines.

Polyarthralgias are the most common sequelae that occur in people who become sick with CHIKV, and this may persist for months or years [25, 26]. This study was the main outcome reported. Acetaminophen and non-steroidal anti-inflammatory drugs (NSAIDs) were the most commonly used treatments to relieve disease symptoms, and their use was previously reported in the literature [27].

In terms of the risk of having high rates of family infection by Chikungunya, in the logistic regression model, the presence of young people, storing water, no garbage collection service and no use nets against mosquitoes were variables that, although presented appropriate theoretical support in the context of infection, could not be statistically demonstrated in the final model. However, the model was able to explain the effect of six of the variables studied; families of Ovejas, households with a family income below the minimum wage, families with a predominance male gender and families with majority of student showed higher RFI rates for Chikungunya. On the other hand, families that used repellent or fumigation, presented low RFI rates for Chikungunya.

The largest RFI in the municipality of Ovejas is consistent with its highest attack rate, the most aggressive epidemiological evolution observed, the largest clinical manifestation and the highest number of health center assistance by its inhabitants. In addition, the area studied in Ovejas showed determining factors that could explain the greater epidemiological consequences of the disease. On the other hand, and despite the fact that all households evaluated in the two municipalities belong to low socio-economic levels, the poorest families, with incomes lower than the minimum income, constituted a higher RFI. Other studies have already shown an increased risk for infectious diseases in low-income families [28].

The high rate of RFI associated with families with greater presence of men, students and children < 15 years can be explained by greater mobility of these outside the home, becoming probably the main transmitters of infection to homes. The high risk of students to the transmission of vector-borne diseases has already been demonstrated [29, 30]. In general, women were more affected than men, which has already been demonstrated by other authors [22], however, The effect of gender within the logistic model has limitations due to the lack of knowledge of this variable in the population that did not suffer the disease.

Repellent use and household fumigation were protective factors with demonstrated effectiveness in the mosquitoe control. Non-use was associated with high rates of family infection. In spite of being related as protection factors, the use of repellents and fumigations were low in the two municipalities, contrary to what was observed in other studies [31, 32]. The low use of repellents and fumigation may be due to the additional costs that they generate, affecting still more subsistence income in affected families.

## Conclusions

In Colombia, the emerging Chikungunya virus produced a serious public health problem, mainly in populations with high vulnerability in health. In these areas, in the municipalities of Ovejas and Corozal, approximately 50% of the population was infected; low rates of attendance at clinic and implications for control of chikungunya were observed. Some families presented much more complex health difficulties; economic problems, coverage problems in health care and lack of preventive measures contributed to the epidemiological complexity of the infection. We built a logistic regression model which suggests that socio-demographic and behavioral factors may be significantly related to odds of infection at the household level. In the future, new changes in public health are expected by the Chikungunya virus, the current presence of Zika and Dengue make the epidemiological scenario of the future more complex.

## Abbreviations

*Ae*: Aedes
CHIKV: Chikungunya virus
ECSA: East/Central/South African Genotype
IIBT: Instituto de Investigaciones Biológicas del Trópico
NSAIDs: Non-steroidal anti-inflammatory drugs
OR: Odds ratio
PAHO: Pan American Health Organization
RFI: Risk of family infection
WHO: World Health Organization
